# Expanding Host Range and Cross-Species Infection of Hepatitis E Virus

**DOI:** 10.1371/journal.ppat.1005695

**Published:** 2016-08-04

**Authors:** Xiang-Jin Meng

**Affiliations:** Department of Biomedical Sciences and Pathobiology, Virginia Polytechnic Institute and State University (Virginia Tech), Blacksburg, Virginia, United States of America; University of Florida, UNITED STATES

## Introduction

Hepatitis E is an important public health disease [1]. Although the mortality rate is less than 1% in the general population, it can reach up to 25% in infected pregnant women. According to the World Health Organization, each year an estimated 20 million infections occur worldwide resulting in >3 million symptomatic cases and 56,600 hepatitis E-related deaths (http://www.who.int/mediacentre/factsheets/fs280/en/). Large explosive waterborne outbreaks of hepatitis E are generally seen in developing countries with poor sanitation conditions, whereas in industrialized countries, sporadic and cluster cases of hepatitis E have been reported. Hepatitis E is a self-limiting acute disease that normally does not go into chronicity. However, recently, chronic hepatitis E has become a significant clinical problem in immunocompromised individuals such as organ transplant recipients [2]. The discovery of animal strains of hepatitis E virus (HEV) [3] that infect across species barriers revolutionizes the way we used to think about this important disease. Hepatitis E is now recognized as a zoonotic disease, and animal reservoirs exist [4]. Herein, I briefly discuss the ever-expanding host ranges, cross-species infection, zoonotic risk, and food safety of HEV.

## What Is Hepatitis E Virus (HEV)?

**Fig 1:**
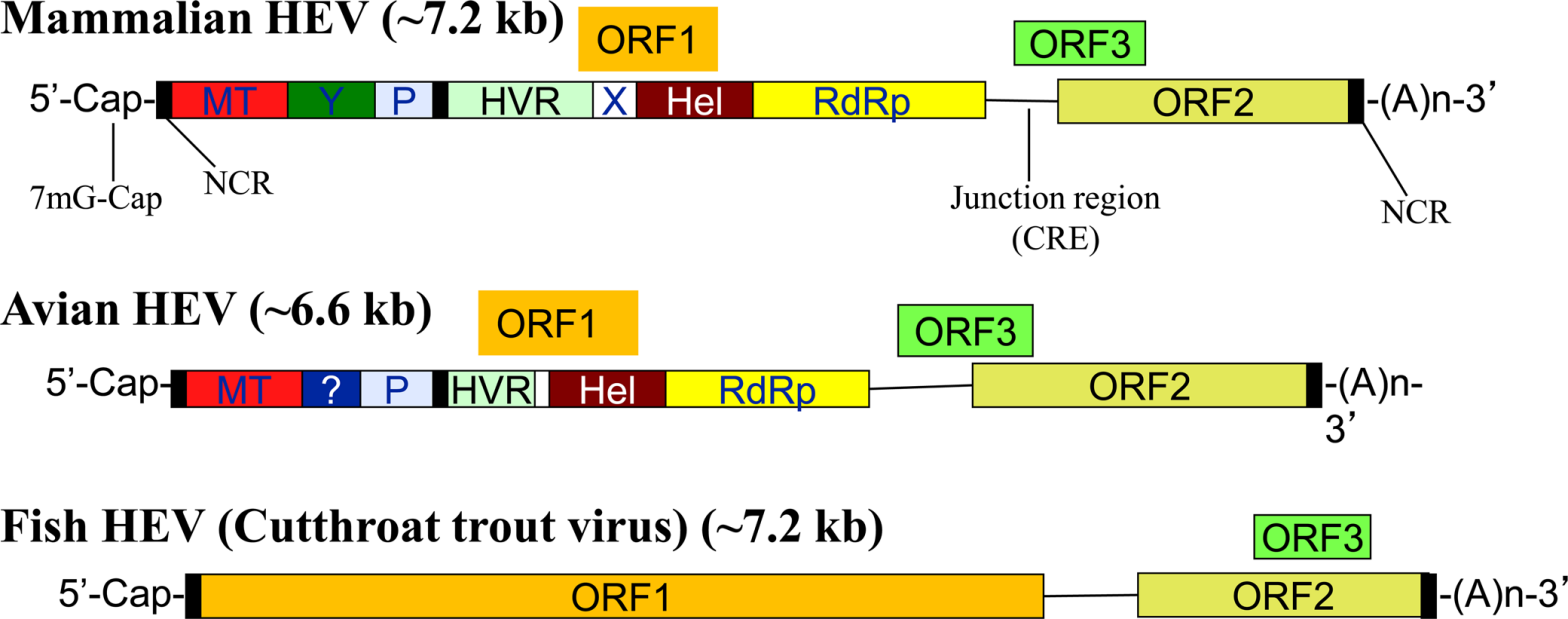
A schematic diagram of comparative genomic organization of mammalian, avian, and fish HEV. The three open reading frames (ORFs) are labeled and shown as boxes. ORF2 overlaps ORF3, but neither overlaps ORF1. ORF1 encodes nonstructural proteins with the putative functional domains within ORF1 indicated inside the box. ORF2 encodes the capsid protein, and ORF3 encodes a small protein that is involved in virus replication. The genome is capped (m7G Cap) at the 5′ end and contains a poly A tail at the 3′ end. There are noncoding regions (NCR) at the 5′ and 3′ ends of the viral genome. There is a junction region between ORF1 and ORF3 for mammalian and avian HEV, which contains a stem-loop structure and a cis-reactive element (CRE). The avian HEV genome is approximately 600 bp smaller than the mammalian and fish HEV. Hel, helicase; HVR, hypervariable region; MT, methytransferase; NCR, noncoding region; P, a papain-like cysteine protease; RdRp, RNA-dependent RNA polymerase; X, macro domain.

HEV is currently classified in the family Hepeviridae [5]. Virions of HEV are non-enveloped, spherical particles of approximately 27–34 nm in size. The genome is a single-strand positive-sense RNA molecule of approximately 7.2 kb and contains three open reading frames (ORFs). ORF1 encodes the non-structural polyprotein, and ORF2 encodes the capsid protein that binds to cell surface heparan sulfate proteoglycans in liver cells. ORF3 encodes a small phosphoprotein with a multifunctional C-terminal region [6]. ORF2 overlaps ORF3, but neither overlaps ORF1 (Fig 1). A cap structure has been identified in the 5′ end of the viral genome and may play a role in the initiation of viral genome replication and protein translation (Fig 1).

Identification of genetically distinct strains of HEV from a number of animal species led to the recent division of the family Hepeviridae into two genera by the International Committee on Taxonomy of Viruses (ICTV): genus *Orthohepevirus* (all mammalian and avian HEV isolates) and genus *Piscihepevirus* (cutthroat trout virus) [5]. There are four species within the genus *Orthohepevirus*: *Orthohepevirus A* (HEV isolates from human, pig, wild boar, deer, mongoose, rabbit, moose, and camel), *Orthohepevirus B* (isolates from chicken), *Orthohepevirus C* (isolates from rat, greater bandicoot, Asian musk shrew, ferret, and mink), and *Orthohepevirus D* (isolates from bat) (Table 1). Within the proposed species *Orthohepevirus A*, at least four genotypes are known to infect humans. Genotype 1 causes large outbreaks in humans in Asia. Genotype 2 includes a Mexican and several African strains and causes large outbreaks. Genotype 3 is associated with sporadic, cluster, and chronic cases of hepatitis E in humans. Genotype 4 is also associated with sporadic cases of hepatitis E in humans.

**Table 1:** Host range of hepatitis E virus infection and zoonotic risk.

Natural animal host	Classification (genus/species, genotypes [gt])	Experimental hosts for cross-species infection	Zoonotic infection in humans
	***Orthohepevirus A***		
Human	gt 1, 2, 3, 4	Non-human primates, pigs (gt 3, 4), rabbits (gt 1, 4), lambs (gt 1), Wistar rats (gt 1)	
Domestic swine	gt 3, 4	Non-human primates, rabbits, Mongolian gerbils (gt 4), Balb/C mice (gt 4)	Yes
Wild boar	gt 3, 4, 5, 6		Yes (gt 3, 4), likely (gt 5, 6)
Deer	gt 3		Yes
Rabbit	gt 3	Pigs	Likely
Mongoose	gt 3		Likely
Camel	gt 7		Yes
Moose	unknown		Not known
	***Orthohepevirus B***		
Chicken	Avian HEV gt 1, 2, 3	Turkeys	No
	***Orthohepevirus C***		
Rat			Unlikely
Ferret			Unlikely
Greater bandicoot			unlikely
Asian musk shrew			unlikely
Mink			unlikely
Bat	***Orthohepevirus D***		No
Cutthroat trout	***Piscihepevirus***		No

## What Are the Host Range and Animal Reservoirs for HEV?

Recent genetic identification of HEV strains from various animal species has significantly broadened the host range and genetic diversity of the virus [4,7]. In addition to humans, HEV has been genetically identified from numerous other animal species including wild and domestic swine, deer, rabbit, mongoose, chicken, camel, rat, ferret, greater bandicoot, Asian musk shrew, mink, moose, and fish (Table 1) [8,9]. The well-characterized animal strains of HEV include genotypes 3 and 4 swine HEV from domestic and wild pigs, genotype 3 rabbit HEV, and avian HEV from chickens. Beside human and swine, genotype 3 HEV strains have also been identified from rabbits, deer, and mongooses. Rats and ferrets each carry HEV-related strains that are genetically distinct from other mammalian and avian HEV (Table 1). The moose HEV clusters within species *Orthohepevirus A* form a distinct clade with a common ancestor to the genotypes 1–6 viruses [9,10]. Cutthroat trout virus resembles mammalian hepeviruses in its genomic organization (Fig 1) despite a low nucleotide sequence identity [11] and represents a separate genus *Piscihepevirus*. The genetic identification of these diverse animal strains of HEV provided opportunities for developing novel, naturally occurring animal models for HEV.

Additionally, serological evidence of HEV infection has also been reported in a number of other animal species, even though the source of seropositivity in these species has not yet been genetically identified. Anti-HEV antibodies are reportedly detected in several ruminant species such as goats, cattle, and sheep, as well as in dogs and cats with no detection of HEV-related sequences. Definitive genetic identification of the sources for HEV seropositivity in these animal species will lead to the discovery of new animal strains of HEV and thus further expansion of HEV host range.

## Can HEV Infect across Species Barriers and Cause Zoonotic Infection?

Genotypes 1 and 2 HEV within the *Orthohepevirus A* species have a rather limited host range and are restricted to humans, because attempts to experimentally infect other species including pigs, rats, and goats with genotypes 1 and 2 human HEV were not successful. Lambs and Wistar rats were reportedly infected by presumably a genotype 1 human HEV, although others failed to infect goats or rats with genotypes 1 and 2 HEV [12]. In contrast, genotypes 3 and 4 HEV have a much broader host range and can infect across species barriers (Table 1). Under experimental conditions, genotypes 3 and 4 swine HEV can readily infect non-human primates and, conversely, genotypes 3 and 4 human HEV infect pigs [13,14]. The avian HEV from a chicken successfully infected turkeys, but failed to infect rhesus monkeys, suggesting that avian HEV is likely not zoonotic. Rabbit HEV infected pigs, and genotypes 1 and 4 human HEV also reportedly infected rabbits [4].

Ample evidences have documented zoonotic infection of genotypes 3 and 4 HEV. The sporadic and cluster cases of human hepatitis E from industrialized countries are mostly caused by the zoonotic genotypes 3 and 4 HEV, and cases of chronic hepatitis E in immunocompromised individuals are definitively linked to zoonotic infection by the genotype 3 HEV as well [2]. Contact exposure to HEV-infected swine leads to an increased risk of zoonotic HEV infection in humans [15]. For example, swine veterinarians in the United States were 1.51 times more likely to be seropositive for HEV antibodies than age- and geography-matched control subjects. Individuals from traditionally major swine states such as Minnesota and Iowa are more likely to be seropositive for anti-HEV antibodies than those from traditionally non-swine states such as Alabama [16]. Clearly, swine are a major reservoir for HEV, and occupational contact with infected swine is a risk factor for zoonotic HEV infection in humans.

Swine is a recognized reservoir for zoonotic HEV infection. However, the presence of numerous other strains of HEV in wildlife and other domestic animal species (Table 1) suggests additional potential mechanisms of zoonotic transmission. For example, deer and rabbits can serve as potential reservoirs for HEV. Zoonotic transmissions of hepatitis E from deer to humans were reported [17]. Field workers who had close contact with wildlife animal species had a significantly higher anti-HEV antibody prevalence than controls [18]. Thus, it is critical to understand the natural history and ecology of HEV infection in order to devise effective preventive and control strategies.

## What Is the Viral Determinant(s) for HEV Cross-Species Infection?

The viral genetic determinant(s) of HEV cross-species infection remains largely unknown. The capsid encoded by ORF2 is the only structural protein and thus is presumed to bind to an unknown cellular receptor and determine host tropism. However, intergenotypic chimeric viruses containing the ORF2 of the zoonotic genotypes 3 or 4 HEV (infecting both humans and pigs) in the genomic backbone of a genotype 1 human HEV (only infecting humans) failed to infect pigs [19]. Similarly, none of the four intergenotypic chimeric viruses with various swapped genomic regions between genotypes 1 and 4 and between genotypes 1 and 3 was able to establish a robust infection in pigs [20]. Therefore, the ORF2 capsid gene does not seem to be important for HEV cross-species infection, and, thus, other genomic regions such as ORF1 are likely involved in the HEV host range. Recently, the adaptation of a unique genotype 3 HEV (Kernow C-1) recovered from a chronically infected patient to propagate in HepG2C3A human hepatoma cells has selected for a rare virus recombinant that contains an insertion of a 171-nucleotide sequence of human ribosomal protein S17 (RPS17) within the hypervariable region of HEV ORF1. When a genotype 1 HEV (only infecting humans) was engineered to contain the RPS17 insertion in its ORF1, the recombinant virus expanded the host range and was able to infect cell lines derived from cows, dogs, cats, chickens, and hamsters [21]. Subsequent studies found that the RPS17 insertion in HEV ORF1 bestows novel nuclear/nucleolar trafficking capabilities to the ORF1 protein of Kernow C-1 HEV and that the lysine residues within the RPS17 insertion, but not nuclear localization of the HEV ORF1 protein, correlate with the enhanced virus replication [22]. More recently, it was demonstrated that a chimeric virus containing the ORF1 gene from a genotype 4 HEV in the backbone of a genotype 1 HEV alters its host cell tropism and infects pig kidney cell line [23], further suggesting a potential role of ORF1 in HEV cross-species infection.

## Is There Any Food Safety Concern about HEV-Contaminated Animal Meat Products?

Food safety associated with HEV contamination in animal meat products is an important public health concern as increasing numbers of food-borne hepatitis E have been reported [24]. Approximately 2% of the commercial pig livers from local grocery stores in Japan, 4% in Germany, 6.5% in the Netherlands, and 11% in the United States tested positive for the zoonotic genotype 3 HEV RNA. Importantly, the contaminating virus in the commercial pig livers remains fully infectious, and cooking the contaminated meat at a temperature similar to a medium-to-rare cooking condition in restaurants did not completely inactivate the virus. Approximately 6% of the sausages sampled at processing and at the point of sale in Spain were also positive for the zoonotic genotype 3 HEV RNA. Sporadic and cluster cases of acute hepatitis E have been linked to the consumption of contaminated raw or undercooked animal meats. In Japan, consumption of wild boar meat, grilled pig entrails, and raw deer meats has been definitively linked to cases of hepatitis E [25]. In France, consumption of pig liver sausages (Figatelli) has been definitively linked to cases of hepatitis E as well [26], and approximately 30% of Figatelli in France tested positive for genotype 3 HEV RNA [27]. Consumption of camel-derived food products (meat and milk) was incriminated for post-transplantation hepatitis E as a camel HEV was identified from the patient [28]. The dissemination of HEV through pork production chains and its associated risk of food-borne transmission are of significant concern. The fact that cases of chronic hepatitis E in immunocompromised individuals are exclusively caused by the zoonotic genotypes 3 and 4, presumably acquired from contaminated meat, underscores the importance of food-borne HEV transmission.

## Perspectives

HEV is an extremely understudied but important human pathogen. Significant progress in HEV research has been made in the past decade, but many important questions remain. The life cycle of HEV is still largely unknown. Identification of a specific cellular receptor for HEV will potentially help establish a more efficient cell culture system for HEV propagation. The ever-expanding host range and identification of new animal reservoirs pose a significant concern for zoonotic HEV infection but also offer new opportunities for developing useful animal models for HEV. The biology, ecology, natural history, and zoonotic potential of these novel animal strains of HEV are still poorly understood. Food-borne cases of hepatitis E in humans are increasingly reported and are likely underestimated in the medical community due to the lack of FDA-approved standardized serological and molecular diagnostic assays for HEV. Chronic hepatitis E associated with genotype 3 or 4 HEV infections has recently become an important clinical problem in organ transplant recipients and other immunocompromised individuals, thus prompting a need for developing effective anti-HEV drugs and vaccines. The recombinant HEV commercial vaccine recently approved for use only in China appears to be promising, but the efficacy of this vaccine and other experimental vaccines against the emerging genetically diversified zoonotic animal strains of HEV is unknown. Development of a vaccine against the zoonotic genotypes 3 and 4 swine HEV, which are highly prevalent in pigs worldwide, would reduce cases of food-borne and zoonotic HEV infections in humans, even though such a vaccine is not a priority for the global pork industry because swine HEV does not cause an economically important pig disease. Such a vaccine would also be useful for high-risk populations such as organ transplant recipients because the vast majority of the chronic hepatitis E cases are caused by the zoonotic genotype 3.
